# Mild Hypoxia Enhances the Expression of HIF and VEGF and Triggers the Response to Injury in Rat Kidneys

**DOI:** 10.3389/fphys.2021.690496

**Published:** 2021-06-25

**Authors:** Yaya Xu, Xiangmei Kong, Jiru Li, Tiantian Cui, Yifan Wei, Jiayue Xu, Yueniu Zhu, Xiaodong Zhu

**Affiliations:** Department of Pediatric Critical Care Medicine, Xinhua Hospital, Affiliated to the Medical School of Shanghai Jiao Tong University, Shanghai, China

**Keywords:** kidney injury, VEGF, HIF-1, chronic hypoxia, vascularization

## Abstract

**Background:**

Hypoxia contributes to a cascade of inflammatory response mechanisms in kidneys that result in the development of renal interstitial fibrosis and subsequent chronic renal failure. Nonetheless, the kidney possesses a self-protection mechanism under a certain degree of hypoxia and this mechanism its adaptation to hypoxia. As the hypoxia-inducible factor (HIF)–vascular endothelial growth factor (VEGF) axis is a key pathway for neovascularization, the activation of this axis is a target for renal hypoxia therapies.

**Methods:**

Sprague–Dawley rats were exposed to normobaric hypoxia and subdivided into three groups, namely group A (21% O_2_), group B (10% O_2_), and group C (7% O_2_). Renal tissue samples were processed and analyzed to determine pathological morphological changes, the expression of HIF, VEGF, inflammation factor and vascular density.

**Results:**

We found that as the duration of hypoxia increased, destructive changes in the kidney tissues became more severe in group C (7% O_2_). In contrast, the increased duration of hypoxia did not exacerbate kidney damage in group B (10% O_2_). As the hypoxia was prolonged and the degree of hypoxia increased, the expression of HIF-1α increased gradually. As hypoxia time increased, the expression of VEGF increased gradually, but VEGF expression in group B (10% O_2_) was the highest. Group C (7% O_2_) had higher levels of IL-6, IL-10, and TNF-alpha. Additionally, the highest vascular density was observed in group B.

**Conclusion:**

These findings suggest that activating the HIF–VEGF signaling pathway to regulate angiogenesis after infliction of hypoxic kidney injury may provide clues for the development of novel CKD treatments.

## Introduction

Oxygen homeostasis is an important factor for maintaining normal energy production. The kidney is one of the most easily damaged organs during hypoxia (Deniz et al., 2017; Wang et al., 2018). An increasing body of evidence suggests that chronic hypoxia can trigger a kidney damage response and may stimulate inflammation in proximal tubule cells, thereby causing irreversible pathological changes. Furthermore, activated interstitial fibroblasts and renal microvascular endothelial cells can lead to the development of tubulointerstitial fibrosis (Fine and Norman, 2008; Liu et al., 2018). Nevertheless, fibrosis development is not always reported. We have observed that renal function can remain normal even in a considerable number of patients with cyanotic congenital heart disease [patients with cyanotic congenital heart disease usually had partial pressure of arterial oxygen (P_*a*_O_2_) of < 90 mmHg)]; these patients usually experience chronic hypoxia for a long time (Zheng et al., 2013). Studies have revealed that hypoxia-inducible factor 1 α (HIF-1α) expression increases gradually after hypoxic injury, and HIF-1α can regulate the expression of downstream genes and increase oxygen delivery to the injured tissue (Majmundar et al., 2010). These findings suggest that there may exist a self-protection mechanism against kidney injury under chronic hypoxic conditions. Research shows that HIF-1α is involved in the mechanisms of adaptation to renal hypoxia (Li et al., 2015). Under normoxic conditions, HIF is degraded by ubiquitin-dependent proteasomes; however, hypoxia stabilizes the expression of HIF, which upregulates the expression of target genes participating in hypoxia adaptation (Semenza, 2014).

In response to the activation of HIF-1α, multiple gene products are expressed [including vascular endothelial growth factor (VEGF)] that counteract hypoxia by promoting angiogenesis (Honda et al., 2019). Research indicates that downregulated VEGF expression and upregulated expression of antiangiogenic factors can lead to the occurrence of dysregulated angiogenesis, eventually culminating in progressive kidney failure (Tanaka et al., 2015).

Notably, hypoxia activates the HIF pathway; however, under severe hypoxic conditions, expression of the HIF pathway is downregulated. A study indicates that the expression of HIF-1α increases under a certain degree of hypoxia (2% O_2_), and on the contrary, it decreases under severe hypoxic conditions (0.5% O_2_) (Guo et al., 2019). It has been suggested that HIF expression correlates with the degree of hypoxia. This theory may be supported by several reports that revealed the existence of decreased HIF-1α expression under severe hypoxic conditions (usually 1% O_2_) at the cellular level (Uchida et al., 2004; Holmquist-Mengelbier et al., 2006). To our knowledge, few studies have addressed the relation between the degree of hypoxia and the expression of HIF in animal models.

A study indicates that IL-6 can regulated VEGF-A mRNA expression, which can promote vascular endothelial cells, smooth muscle cell mitosis, vascular permeability, and angiogenesis (Ferrara et al., 2003; Li et al., 2008). IL-6 can drive a hypoxic response by activating the PI3K/AKT/NF-kB and PI3K/AKT/mT0R pathway, leading to increased expression levels of VEGF (Li et al., 2016; Elhadidy et al., 2021). Pro-inflammatory cytokines secreted from endothelial cells, which function in autocrine or paracrine loops, will further activate endothelial cells, leading to angiogenesis (Tolentino et al., 1996).

Therefore, in this study, we hypothesized that (i) kidneys could tolerate a certain degree of hypoxia and (ii) under these conditions, the lack of oxygen might lead to the activation of HIF-1α and expression of inflammatory cytokines, thereby promoting angiogenesis.

## Materials and Methods

### Animals and Experimental Procedures

Thirty 5-week-old male Sprague–Dawley (SD) rats [weighing 146.9 ± 6.8 g (mean ± SD); Xinhua Hospital Experimental Animal Center] were used in this study. Traditionally, for the chronic hypoxia model, rats were subjected to hypoxia (10% O_2_) for 2 weeks (Corno et al., 2002). Oxygenation target (P_*a*_O_2_ 90–60 mmHg) was achieved by adjusting fraction of inspiration O_2_ (FiO_2_), and for a high survival rate and low P_*a*_O_2_ level, the minimum O_2_ concentration we measured in our study was 7% O_2_.

The rats were randomly distributed into three groups according to the inhalation of different concentrations of oxygen (each group comprised 10 animals): 21% O_2_ in group A, 10% O_2_ in group B, and 7% O_2_ in group C.

**FIGURE 1:**
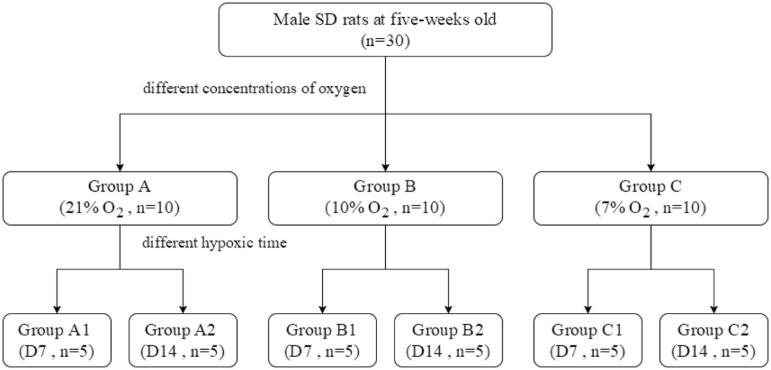
The design of animal experiments.

Each group was further divided into two subgroups according to hypoxia duration (1 or 2 weeks) (Figure 1). The rats were housed in a room with light control (12-h light/12-h dark), temperature control (22 ± 2°C), and 40–45% humidity and had free access to laboratory water and feed. This study was conducted in strict accordance with the recommendations of the guidelines of the Institutional Animal Care and Use Committee of Xinhua Hospital Affiliated with Shanghai Jiao Tong University School of Medicine (Shanghai, China), and the experimental protocol was approved by the Ethics Committee of the same institution.

### Chronic Normobaric Hypoxia Exposure

**FIGURE 2:**
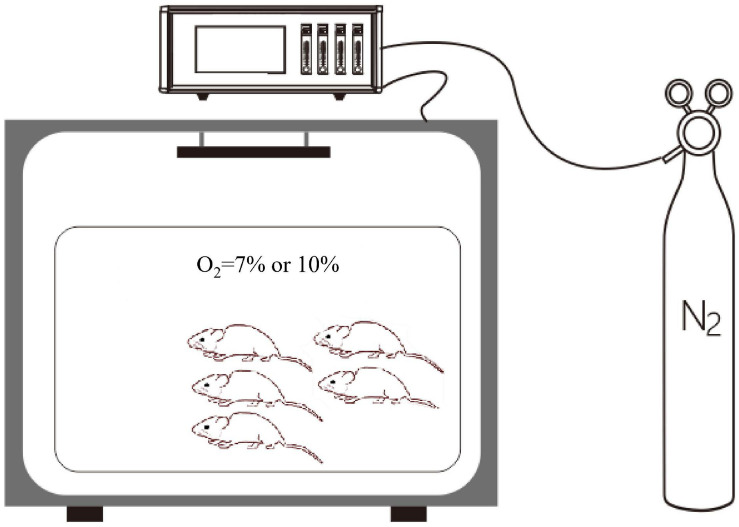
The normobaric hypoxic chamber. A proportional integral differential controller is used for the regulation of pressure and for the control of the solenoid valve. The oxygen concentration was regulated by using an oxygen controller with a nitrogen generator. The oxygen concentration range and accuracy were 0–25% and 0.1%, respectively.

Rats in the control group were exposed to 21% O_2_, while rats in the experimental groups were exposed to different degrees of hypoxia for at least 1 week. All rats in the hypoxic groups were placed in a normobaric hypoxic chamber (ProOx-100, Shanghai TOW Intelligent Technology Company, China). Briefly, the control normoxia group was exposed to room air, while the hypoxia groups were caged in a hypoxic chamber (D × W × H: 56 × 40 × 32 cm; *n* = 5 per cage), as presented in Figure 2. At the end of the treatment [day 7 (D7) or D14], the rats were anesthetized with 10% chloral hydrate (350 mg/kg, i.p.). After successful anesthetization, the rats were placed in the supine position and fixed on an operating table. A midline ventral incision was performed to isolate the abdominal aorta, a polyethylene catheter was inserted into the abdominal aorta, and a pressure transducer was connected to a data acquisition system (Medlab-TA, Nanjing Medease Science and Technology) for blood pressure measurement.

Blood pressure was measured through the abdominal aorta, and blood samples were collected for testing. The rats were then euthanized by exsanguination. Both kidneys were excised for analysis. One specimen was flash-frozen and immediately stored in a liquid nitrogen container for conducting western blot analysis, while the other sample was soaked in 4% paraformaldehyde solution for fixation prior to the performance with staining and immunohistochemical examination. Tissue sections were observed under the Nikon Eclipse E600 microscope, and non-overlapping images were photographed using the Nikon DS-Ri1 digital camera.

### Tissue Processing and Immunohistochemical Analysis

Each kidney specimen was fixed in a 4% paraformaldehyde solution for 24 h at room temperature, and the maximum cross-section, including the cortex, medulla, and papilla, was selected for continuous sectioning after completion of paraffin embedding. The embedded tissue fragments were cut into slices of 3 μm thickness. Histological staining was performed with hematoxylin and eosin (H&E), Periodic acid-Schiff (PAS) staining and Masson’s trichrome staining. SP immunohistochemistry was used to detect the expression of HIF-1α and VEGF in renal tissues. The following primary antibodies were used for overnight incubation: anti-rat HIF-1 alpha (Novus Biological, Littleton, CO) and anti-rat anti-VEGF (Santa Cruz Biotechnology, Dallas, TX, United States). Three renal sections for samples obtained from each animal were selected, and five high-power fields (SP × 200) were randomly chosen in each section. HIF-1α and VEGF expression levels were quantified according to the formula “immunohistochemical mean density = integrated optical density (IOD) sum/area in three randomly selected microscopic fields for each slide” using the Image-Pro Plus 6.0 (Media Cybernetics, United States).

### Immunofluorescence Assay

Paraffin-embedded kidney tissue sections were dewaxed using xylene and rehydrated through usage of a graded series of alcohol solutions, with completion of the final rehydration steps using water. Slides were incubated with 3% hydrogen peroxide for 10 min to block endogenous peroxidase activity and then kept in 10 mM sodium citrate buffer (pH 6.0) to recover antigens. Slides were incubated overnight at 4°C with the following primary antibodies: a rabbit polyclonal anti-α-SMA antibody (1:200 dilution; Abcam) and a goat polyclonal anti-CD31 antibody (1:200 dilution; Santa Cruz Biotechnology). The slides were probed with fluorescently labeled secondary antibodies as follows: a green fluorescent cyanine dye for α-SMA [DyLight 488–conjugated donkey anti-rabbit IgG (H + L) antibody; Biogot Technology, Nanjing, China] at a dilution of 1:200; a red fluorescent cyanine dye for CD31 [TRITC-conjugated mouse anti-goat IgG (H + L) antibody; Biogot Technology, Nanjing, China], for 1 h at room temperature in the dark. After rinsing of the slides with Tris-buffered saline containing 0.05% of Tween 20, the tissue sections were incubated with 4′,6-diamidino-2-phenylindole (DAPI) to perform nuclear staining. Lastly, a fluorescence-quenching solution was added to the mixture. Three sections were randomly selected from each group, and five high-power visual fields were randomly selected in each section and examined. Vascular density was evaluated at 400× magnification.

### Western Blot Analysis

Frozen kidney tissues were added to 500 μL of chilled lysis buffer, followed by centrifugation at 4°C and storage at −80°C. Protein concentration was determined by performing the Bradford method. The following primary antibodies were used: anti-HIF-1a (1:1,000, H6536, Sigma-Aldrich, St. Louis, MO, United States), mouse monoclonal anti-VEGF (1:500; SC-57496, Santa Cruz Biotechnology), and anti-β-actin (1:1,000, A2228, Sigma-Aldrich). Protein expression levels were normalized to those of β-actin and quantified by using the ImageJ software, v1.52 (National Institutes of Health, Bethesda, MD, United States). The relative expression levels of proteins in each group are represented by the ratio of the gray density value of the target protein to that of β-actin.

### Inflammatory Cytokines by Enzyme Linked Immunosorbent Assay

The concentrations of IL-6 (SEA079Ra), IL-10 (SEA056Ra) and TNF-α (SEA133Ra) in rats’ serum were determined using ELISA kits (Cloud-Clone Corp., United States). Protocols of each ELISA kit were followed in this experiment.

### Statistical Analysis

The mean ± standard deviation (SD) values were calculated for all data. The comparisons between groups were performed by using one-way analysis of variance (ANOVA), while differences between two independent groups were analyzed by using the independent-sample *t*-test. All statistical analyses were performed using SPSS version 23 [IBM Corp., Released (2015), IBM SPSS Statistics for Windows; Version 23.0; IBM Corp., Armonk, NY, United States]. Differences were considered significant when the two-tailed *P*-value was less than 0.05.

## Results

### Comparison of Blood Parameters of Rats Among the Groups

No deaths occurred during the experimental period. Red blood cell (RBC) counts and levels of hemoglobin (Hb) and hematocrit (Hct) in hypoxia model rats (groups B and C) were notably higher than those of normal control rats (Group A). On D7 and D14, significant differences were observed in the RBC count and levels of Hb and Hct between groups A and C (*P* < 0.05). PaO_2_ differed among the groups as follows: 98.0 ± 1.0 mmHg in group A, 88.8 ± 12.5 mmHg in group B, and 80.8 ± 20.2 in group C. The difference between the groups was statistically significant (*P* < 0.05). Marked differences were observed between D7 and D14 regarding arterial partial pressure of carbon dioxide (PaCO_2_) and buffer excess (BE). Levels of serum creatinine (SCr) were significantly higher in group C than those in groups A and B (*P* < 0.05). Moreover, in group C, the levels of SCr were significantly higher on D14 than those on D7 (Table 1).

**TABLE 1:** The results of blood analysis performed for rats of the three groups.

**Characteristics**	**Group A**		***P-*value**	**Group B**		***P-*value**	**Group C**		***P-*value**
	**A1 (*n* = 5)**	**A2 (*n* = 5)**		**A1 (*n* = 5)**	**A2 (*n* = 5)**		**A1 (*n* = 5)**	**A2 (*n* = 5)**	
**Blood routine examination**									
RBC (10^9/L)	5.5 ± 0.7	5.1 ± 0.4	0.856	6.8 ± 0.3	6.9 ± 0.4	0.227	8.9 ± 0.2 ^*A*1 B1^	8.9 ± 0.8^ A2 B2^	0.281
HB (g/L)	118.2 ± 7.2	111.4 ± 8.8	0.310	157.6 ± 6.7	169.8 ± 5.4	0.143	195.0 ± 4.2^ A1^	203.8 ± 7.7^ A2 B2^	0.221
Hct	0.38 ± 0.01	0.40 ± 0.03	0.849	0.52 ± 0.05	0.56 ± 0.05	0.222	0.66 ± 0.02^ A1^	0.66 ± 0.04 ^*A*2^	0.191
WBC (10^9/L)	3.8 ± 0.1	4.0 ± 0.4	0.513	5.01 ± 1.0	4.1 ± 0.9	0.275	4.9 ± 1.6	3.0 ± 1.6	0.127
PLT (10^9/L)	747.3 ± 42.2	907.5 ± 179.5	0.275	697.1 ± 142.0	836.0 ± 105.5	0.400	655.0 ± 17.0	408.3 ± 46.5	0.050
**Blood gas analysis**									
pH	7.4 ± 0.1	7.4 ± 0.1	0.819	7.4 ± 0.1	7.4 ± 0.2	0.851	7.4 ± 0.1	7.4 ± 0.1	0.854
PaO_2_ (mmHg)	97.4 ± 1.1	98.0 ± 1.0	0.590	90.5 ± 18.1	88.8 ± 12.5^ A2^	0.802	86.0 ± 14.6^ A1^	80.8 ± 20.2^ A2^	0.402
SaO_2_ (%)	97.8 ± 1.3	99.2 ± 0.8	0.606	95.9 ± 2.4	95.8 ± 1.5	0.925	95.6 ± 2.3	94.2 ± 5.4^ A2^	0.078
PaCO_2_ (mmHg)	41.5 ± 8.5	41.2 ± 7.0	0.794	30.7 ± 4.7	41.6 ± 5.0^ A2^	0.008	27.6 ± 5.1^ A1^	28.9 ± 9.3^ A2^	0.669
BE (mmol/L)	2.2 ± 1.4	2.6 ± 1.0	0.259	−8.6 ± 2.5^ A1^	3.2 ± 1.3	0.004	−9.0 ± 3.3^ A1^	−13.2 ± 7.0^ A2^	0.850
**Renal function index**									
SCr (μmol/L)	21.0 ± 0.7	23.2 ± 0.8	0.034	25.6 ± 0.4	23.0 ± 2.1^ A2^	0.051	27.0 ± 3.2^ A1^	32.2 ± 3.2^ A2^	0.002

*21% O_2_ for A group; 10% O_2_ for B group; 7% O_2_ for C group. Each group was further divided into 2 subgroups according to different hypoxic time (A1 = D7; A2 = D14). RBC, Red Blood Count; HB, Hemoglobin; Hct, Hematocrit; WBC, White Blood Cell; PLT, Platelets; PaO_2_, Arterial Partial Pressure of Oxygen; SaO_2_, Arterial Oxygen Saturation; PaCO_2_, Arterial Partial Pressure of Carbon Dioxide; BE, Buffer Excess.*

*A two-tailed P < 0.05 was considered statistically significant; compared with group A1, ^*A*1^P < 0.05; compared with group A2, ^*A*2^P < 0.05; compared with group B1, ^*B*1^P < 0.05; compared with group B2, ^*B*2^P < 0.05, respectively.*

### Rat Growth and Kidney Weight

**FIGURE 3:**
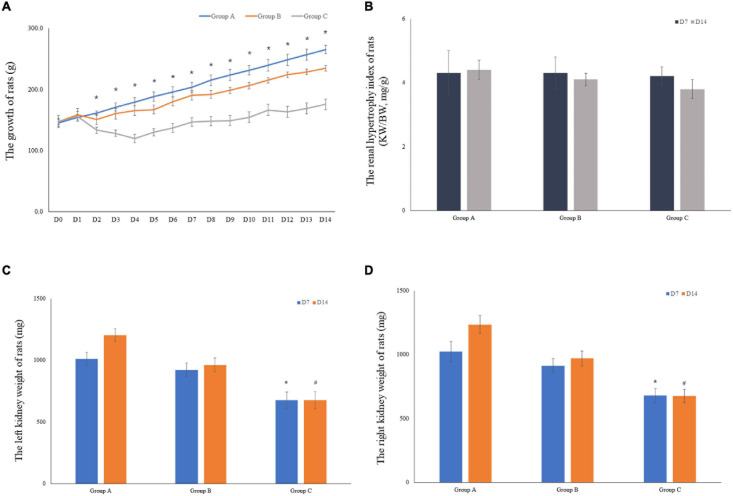
The growth of rats and the weight of rat kidneys. **(A)** The mean body weight of the rats was determined on a daily basis for 14 consecutive days (10 rats were included in the analysis for the first 7 days, and 5 rats were included for the remaining 7 days). **(B)** The renal hypertrophy index of rats on D7 (*n* = 5) and D14 (*n* = 5). **(C)** The mean left kidney weight of the rats was determined on D7 (*n* = 5) and D14 (*n* = 5). **(D)** The mean left kidney weight of the rats was measured on D7 (*n* = 5) and D14 (*n* = 5). *P < 0.05 vs. group A1; ^#^*P* < 0.05 vs. group A2, respectively.

The weights of 10 rats were included in the analysis for the first 7 days. Five rats were euthanized on D7 in both groups. Therefore, the weights of the remaining five rats were considered in the analyses for the remaining 7 days. When the experiment was commenced, a gradual decrease in the weight of rats in groups B and C was observed. By contrast, weight gain of rats in group B occurred at a lower rate compared to group C, and most of them recovered their weight loss in the first 2 days. Group C showed slightly increased body weight (175.8 ± 7.0) on D14 as compared to the initial body weight (147.5 ± 5.8); however, in comparison with the other groups, the body weight of group C rats was lower. The body weight of rats in group A on D14 was documented, followed by that of groups B and C. Particularly, the body weight of rats in group C showed significant differences from group A on D2 to D14 (Figure 3A). An index of renal hypertrophy was estimated by comparing the wet weight of the left kidney to the total body weight; no statistically significant difference was found among the three groups (*P* > 0.05). In groups B and C, the renal hypertrophy index on D14 was lower than that on D7, and the decreasing trend in group C was evident (Figure 3B). The kidney weight was lower in group C than that in groups A and B, and the difference was statistically significant (*P* < 0.05). The kidney weight in group C on D14 was slightly higher than that on D7 but increased less remarkably in comparison with groups A and B (Figures 3C,D).

### General Features and Morphological Traits

**FIGURE 4:**
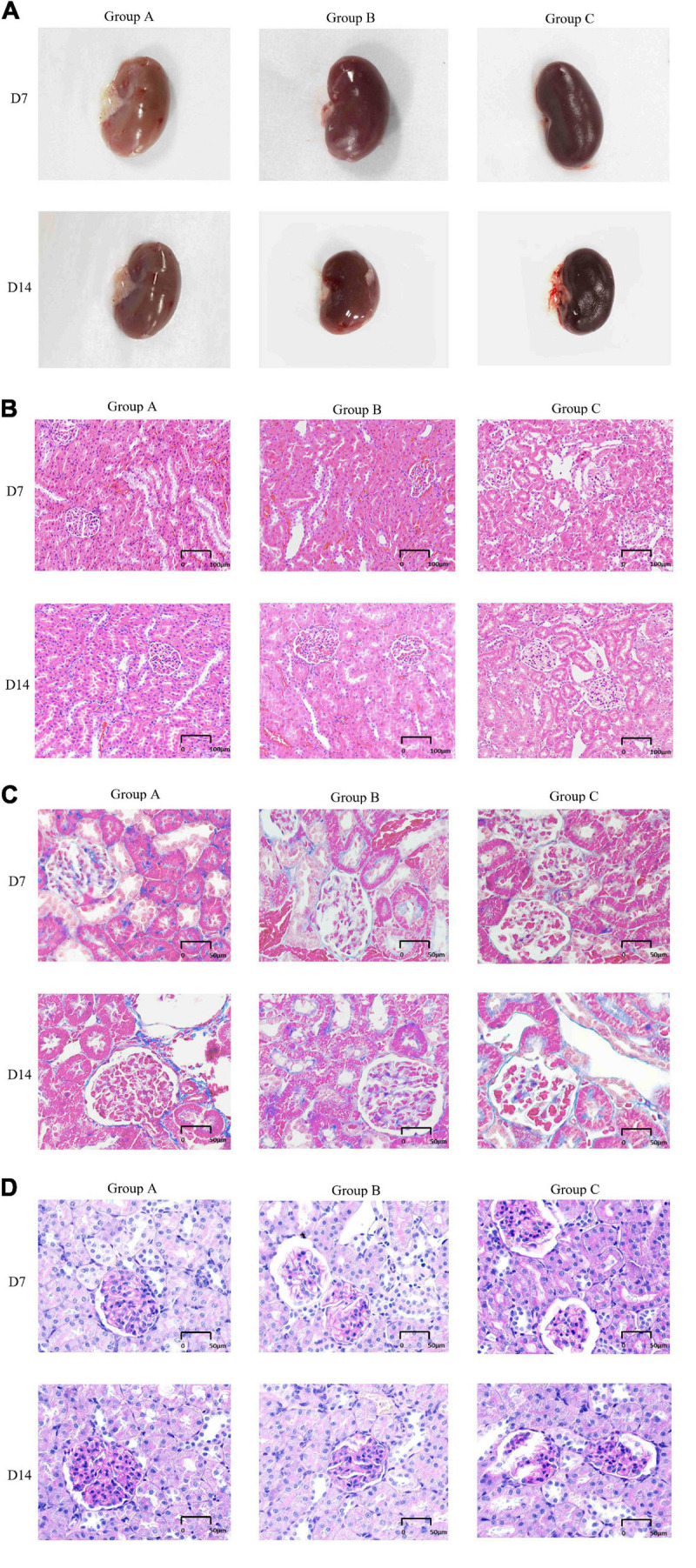
General features and morphological traits in renal tissues obtained from animals subjected to normoxic and hypoxic conditions. **(A)** Gross view of a kidney. **(B)** A histological section of kidney tissue. The histological examination was performed at 20 × magnification (scale bar = 100 μm). **(C)** Periodic acid-Schiff staining (40 × magnification, scale bar = 50 μm). **(D)** Masson’s trichrome staining (40× magnification, scale bar = 50 μm).

The external gross appearance of kidneys in groups A and B was normal, whereas that in group C was suggestive of moderate to severe injury. The kidneys of rats in groups A and B resembled broad beans; they were mobile and soft and had a smooth surface with clear boundaries. The rat kidneys in group C appeared dark in color and had a rough and wrinkled surface (Figure 4A). H&E staining, Masson staining and PAS staining were performed to determine morphological characteristics. The kidneys of rats in group A exhibited no pathological abnormalities, renal glomeruli were found to be preserved, and the renal tubules showed normal appearance. In group B, on D7, the kidneys presented with slight glomerular hypertrophy and mild infiltration of inflammatory cells in the renal interstitium; however, the pathological changes diminished on D14. Local kidney damage was present in rats in group C. Kidney tubule damage markedly increased on D14 when compared with that observed on D7 (Figure 4B). In group C, the glomerular basement membranes were slightly thickened, the mesangial matrix was mildly hyperplastic and disseminated microthrombosis in capillaries was observed by Masson’s trichrome staining. There were some red blood cells in the renal glomerulus and tubules in groups A and B according to pathological examinations (Figure 4C). PAS staining showed that the basement membrane of glomerular capillaries in the kidney from the rat in group C thickened, the glycogen and mesangial matrix increased in the mesangial region, and the mesangial area widened (Figure 4D).

### Renal Expression of HIF-1α and VEGF

**FIGURE 5:**
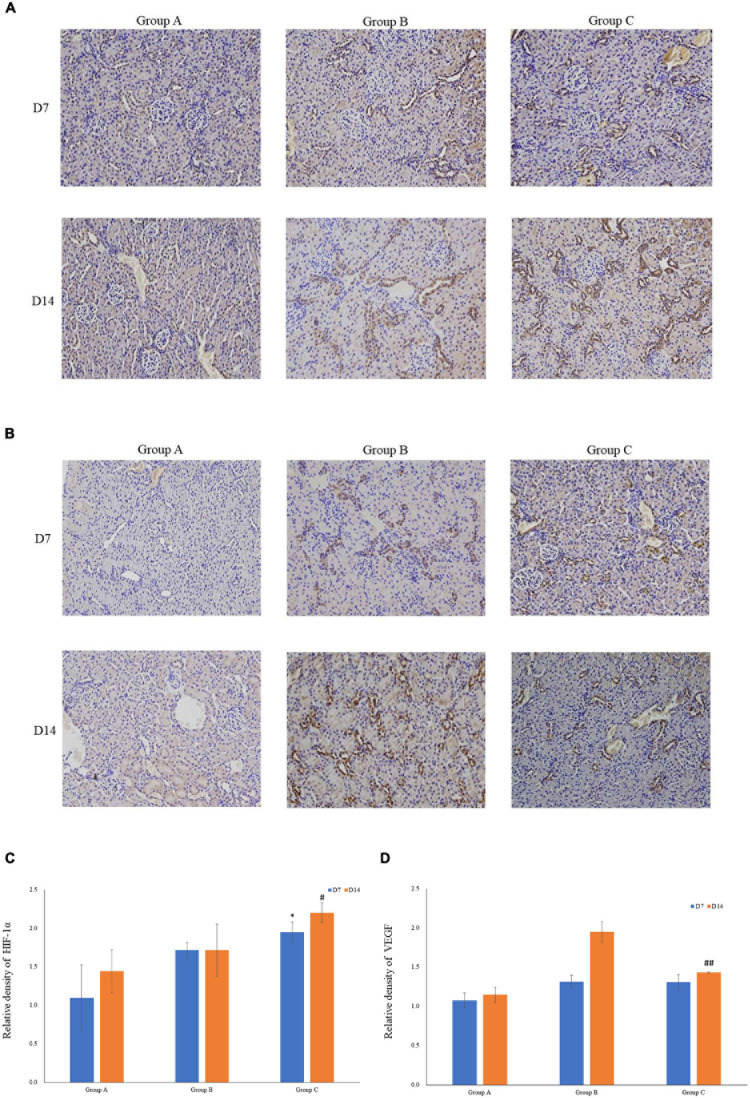
The expression levels of HIF-1α and VEGF in rat kidneys. **(A)** Localization of HIF-1α expression in kidneys (immunohistochemistry; original magnification: ×200). **(B)** Localization of VEGF expression in kidneys (immunohistochemistry; original magnification: ×200). **(C)** Relative levels of HIF-1α. **(D)** Relative levels of VEGF. ^∗^*P* < 0.05 vs. group A1; ^#^*P* < 0.05 vs. group A2; ^##^*P* < 0.05 vs. group B2, respectively.

**FIGURE 6:**
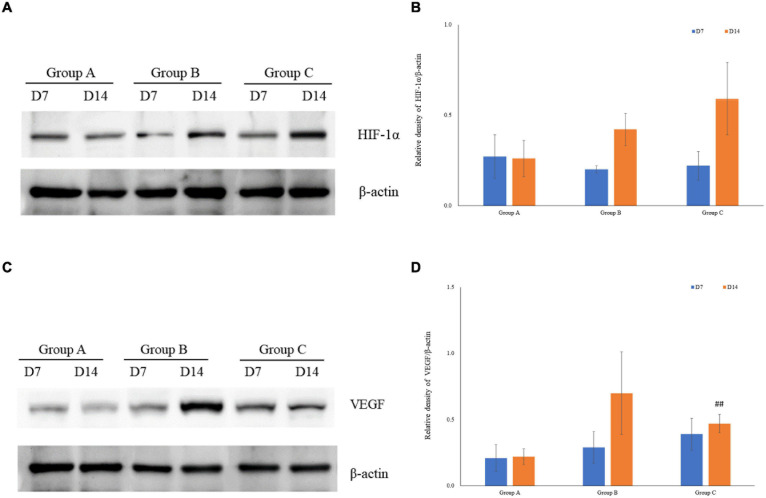
The expression of HIF-1α and VEGF measured by western blotting using rat kidney samples. **(A)** The expression of HIF-1α in rat kidneys. **(B)** Quantitative analysis of HIF-1α by western blotting using rat kidney samples **(C)** The expression of VEGF in rat kidneys. **(D)** Quantitative analysis of VEGF in rats’ kidneys by western blotting. ^##^*P* < 0.05 vs. group B2.

VEGF and HIF-1α expression in kidney tissues was examined by conducting the immunohistochemical method. The expression of HIF-1α demonstrated time-dependent increases in groups B (1.7 ± 0.1 at D7; 1.7 ± 0.3 at D14) and C (1.9 ± 0.1 at D7; 2.2 ± 0.1 at D14), and was significantly higher in group C than that in group A (1.1 ± 0.4 at D7; 1.4 ± 0.3 at D14), but no significant difference was found between groups A and B (*P* > 0.05, Figures 5A,C). Mean density of VEGF in group C was significantly lower than that in the other groups, and the level of VEGF in group A on D7 was the lowest (1.1 ± 0.1). The expression of VEGF in all three groups on D14 was significantly higher than that on D7, especially in group B. Of note, in spite of the highest HIF-1α expression in group C on D14, the expression of VEGF on D14 was lower in group C than that in group B (Figures 5B,D). The western blot analysis results were consistent with the immunohistochemical results (Figures 6A–D).

### Renal Vascular Density

**FIGURE 7:**
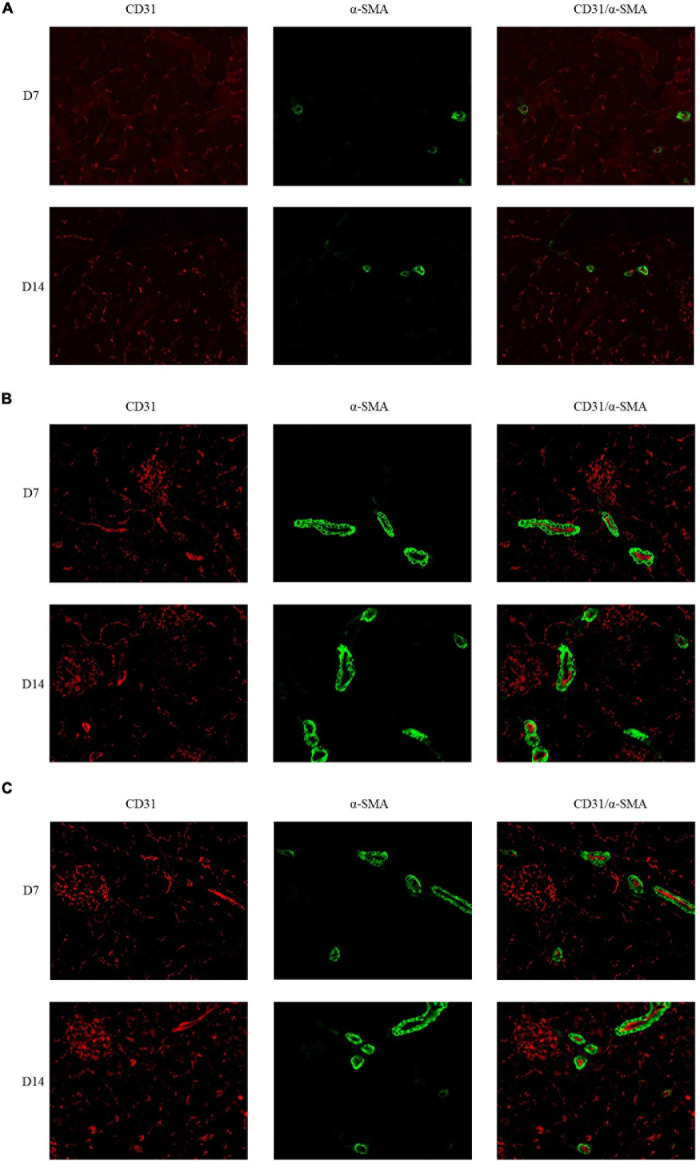
Double immunofluorescence labeling of CD31 (red) and α-SMA (green) in rat kidneys; nuclei are stained with DAPI (blue). **(A)** Mature blood vessel density of rats in the 21% O_2_ group. **(B)** Mature blood vessel density of rats in the 10% O_2_ group. **(C)** Mature blood vessel density of rats in the 7% O_2_ group. Original magnification: ×400.

**FIGURE 8:**
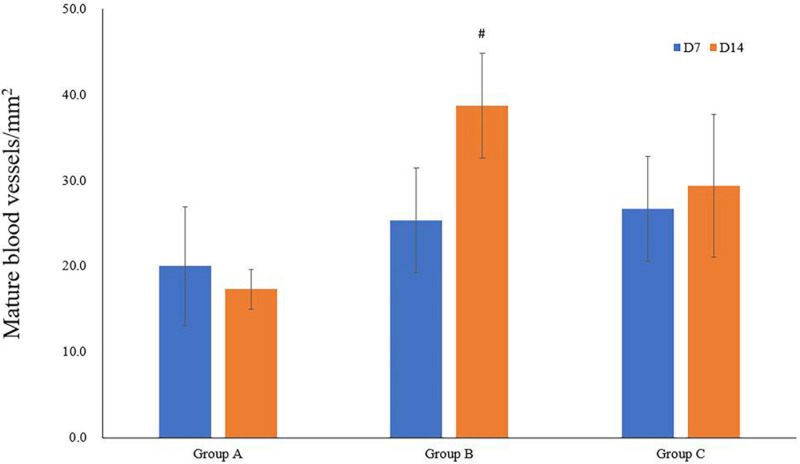
Quantitative analysis of mature blood vessel densities. ^#^*P* < 0.05 vs. group B1.

The immunofluorescence costaining for CD31 and α-SMA revealed that the amount of mature blood vessels in both groups A and B increased compared to that in group C (Figure 7). The quantitative data analysis of the mature blood vessel density confirmed that this parameter had significantly higher values in groups A (26.7 ± 6.1 at D7; 29.3 ± 8.3 at D14) and B (25.3 ± 6.1 at D7; 38.7 ± 6.1 at D14) than those in group C (20.0 ± 6.9 at D7; 17.3 ± 2.3 at D14). Group B rats had the highest mature blood vessel density on D14, which was statistically greater relative to that in rats of group C (*P* < 0.05, Figure 8).

### Pro-inflammatory Indices in Plasma

The effects of hypoxia on IL-6, IL-10, and TNF-α are shown in Table 2. Significant increase in the level of IL-6, IL-10, and TNF-α was observed in group C on D14 when compared with groups A and B (*P* < 0.001 and *P* < 0.05, respectively).

**TABLE 2:** The results of blood analysis performed for rats of the three groups.

**Characteristics**	**Group A**		***P-*value**	**Group B**		***P-*value**	**Group C**		***P-*value**
	**A1 (*n* = 5)**	**A2 (*n* = 5)**		**A1 (*n* = 5)**	**A2 (*n* = 5)**		**A1 (*n* = 5)**	**A2 (*n* = 5)**	
IL-6	11.5 ± 0.8	11.7 ± 1.2	0.847	11.4 ± 0.02	11.4 ± 0.3	0.712	11.5 ± 0.4	12.6 ± 0.4 ^*B*2^	0.002
IL-10	16.5 ± 0.6	17.5 ± 0.9	0.070	16.2 ± 0.5	16.8 ± 0.6	0.117	16.6 ± 0.5	29.2 ± 1.0^ B2^	<0.001
TNF-α	5.9 ± 0.2	5.5 ± 0.6	0.252	4.7 ± 0.9	4.3 ± 0.4	0.402	4.7 ± 0.7 ^*A*1^	9.7 ± 0.4^ B2^	<0.001

*21% O_2_ for A group; 10% O_2_ for B group; 7% O_2_ for C group. Each group was further divided into 2 subgroups according to different hypoxic time (A1 = D7; A2 = D14). Compared with group A1, ^*A*1^P<0.05; compared with group B2, ^*B*2^P < 0.05, respectively.*

## Discussion

This work indicates that an increase in blood vessel density markedly attenuates the progression of renal fibrosis, when the intervention is initiated during early stages of renal disease (Tanaka et al., 2015; Ferreira de Almeida et al., 2019). The main finding of the current study is that subjection to normobaric hypoxia (10% O_2_) for 2 weeks induced subtle renal injury and that the kidney could repair itself under these conditions. On D14, the degree of kidney damage was lower as compared to that observed on D7 and was accompanied by changes in HIF-1α and VEGF expression. Few studies have shown that chronic mild hypoxia can cause elicitation of adaptive responses in the mammalian body (Samanta et al., 2017). When oxygen availability is scarce, the body can easily undergo a series of free-radical metabolism changes; for example, kidneys produce EPO in response to hypoxia, and EPO in turn stimulates the production of red blood cells in the bone marrow (Chen and Sang, 2016). The most recent studies indicate that hypoxia can upregulate the expression of HIFs, which in turn affect the expression of a number of target genes, thereby enhancing survival under conditions of low energy demand in an environment of low oxygen supply (Choudhry and Harris, 2018; Zhang et al., 2018). A study comparing the levels of Hb in athletes between a normobaric hypoxia group and a hypobaric hypoxia group showed that Hb content in the hypobaric hypoxia group was significantly higher than that in the normobaric hypoxia group (*P* < 0.001; Hauser et al., 2016). Therefore, a normobaric hypoxia model was used in the present study to assess the state of kidneys.

### Renal Morphological Alterations

The response of renal tubular epithelial cells to hypoxia is a fundamental pathogenic mechanism implicated in the response to chronic hypoxic kidney injury. In our model, we performed H&E, PAS, and Masson staining to examine possible pathological morphological alterations in the kidneys. The results indicate inflammatory cell infiltration, tubular dilation, and structural disorder on D7 in group C rats. As the duration of hypoxia increased, the destructive changes in the kidney tissues became more severe in group A rats. On D14, parts of the renal tubules collapsed and were obliterated by lumen necrosis. Nonetheless, only a few tubules showed dilation, while the area that surrounded them showed a normal appearance, and renal glomeruli exhibited a normal structure. Only minor renal damage, especially in renal tubules, was observed in group B rats. Increasing the duration of hypoxia did not exacerbate kidney damage. In group B, on D14, renal morphological alterations were slightly better than those on D7. We speculate that the mechanism of renal self-repair is related to the degree of hypoxia. Under severe hypoxic conditions, renal injury is aggravated over time, whereas the kidneys can protect themselves against mild hypoxia. Hypoxia damages renal tubular epithelial cells, and this damage activates the inflammatory cascade response and contribute to the pathogenesis of renal fibrosis. Next, renal fibrosis aggravates hypoxic conditions and forms a vicious circle (Tanaka et al., 2014). In contrast, many investigators have proved that renal tubular epithelial cells can repair and regenerate themselves after infliction of kidney injury (Yang et al., 2010). Animal studies have revealed that renal tubular epithelial cell proliferation peaks at 3 days postinjury and decreases with time to return to near-normal levels by 6 weeks postinjury (Yang et al., 2010). Another study suggests that hypoxia induces the expression of VEGF and activates angiogenesis, thereby alleviating kidney injury (Wang et al., 2015).

### Increased HIF-1α Levels

HIF is a heterodimeric transcription factor that is represented by three different functional HIF-α complexes (HIF-1α, HIF-2α, and HIF-3α), among which the HIF-1α protein plays an important role in hypoxia. The expression of HIF is regulated by prolyl hydroxylases (PHD), wherein PHD activity inhibits the activation of HIF (Semenza, 2014). Few researchers have confirmed an important role played by HIF in renal development, indicating that HIF-1α is associated with numerous biological processes, with evident roles observed during early embryonic development and extended roles in adulthood (Gunaratnam and Bonventre, 2009; Kobayashi et al., 2017). In the present study, we performed immunohistochemical and western blot analyses to evaluate HIF-1α expression. The results showed that as hypoxia was prolonged and as the degree of hypoxia increased, the expression levels of HIF-1α increased gradually. Our data also indirectly confirm the observations reported by Poitz et al. (2014), whose *in vitro* study suggested that the expression of HIF-1α was correlated with the duration and severity of hypoxia. Despite the protective effect of HIF against renal injury, evidence supports the view that chronic overactivation of HIF induces organ injury. Zuolin (2019) have reported that the effects of HIF-1α on renal tubulointerstitial fibrosis in CKD mice are concentration-dependent and bidirectional. These findings indicate that low to moderate activation of HIF-1α inhibits the development of renal tubulointerstitial fibrosis in CKD mice, whereas hyperactivation of HIF-1α triggers the TGF-β signaling pathway, resulting in the development of tubulointerstitial fibrosis (Zuolin, 2019). This phenomenon might explain why HIF expression was higher and kidney injury was more severe in group C. Research has revealed that long-term overactivation of HIF-1α is involved in angiotensin II–induced *IL-6* mRNA expression and aggravated renal injury (Zhu et al., 2011).

### Increased VEGF Levels

VEGF is a major regulator of angiogenesis and is mainly produced by macrophages, tumor cells, and fibroblasts; VEGF expression is mediated by HIF-1α (Tanaka et al., 2015). Several studies have shown that hypoxia promotes the proliferation and migration of vascular endothelial cells by changing the expression of VEGF. One such study indicates that mice lacking VEGF receptor (VEGFR) possess markedly reduced numbers of endothelial cells and blood vessel formation is not observed (Carmeliet et al., 1996). These observations suggest that the VEGF–VEGFR signaling pathway is important for angiogenesis regulation. Our immunohistochemical data and western blot results indicated that VEGF expression was significantly higher in the hypoxic groups than that in the normoxic group, especially in group B. Moreover, the highest expression of HIF-1α in our study was observed in group C, as already mentioned in the previous paragraphs.

In theory, hypoxia exposure activates HIF-1α, thereby increasing VEGF expression. Nevertheless, the expression of VEGF can be affected by several factors as follows: (a) AP-1, SP-1, and STAT3 can induce VEGF expression by binding to the respective gene promoters to initiate and activate the transcription of the *VEGF* gene directly (Josko and Mazurek, 2004); (b) the binding of SDF-1α to CXCR4 eventually causes increased expression of VEGF via the phosphoinositide-3 kinase AKT signaling pathway (Carretero-Ortega et al., 2010; Mohajer Ansari et al., 2020); and (c) insulinlike growth factor 1, CEACAM-1, and platelets can also regulate VEGF expression (Lima et al., 2012; Kleefeldt et al., 2019). In our study, the number of concentrated platelets was higher in group B than that in group C, which may explain the upregulated expression of VEGF observed therein. Few investigators have reported that incubation of platelets with thrombin *in vitro* results in the release of considerable amounts of VEGF (Weltermann et al., 1999; Merigo et al., 2018). The fact that the number of platelets was observed to be less in group C compared to that in group B may be explained by an increased erythropoietin production and erythroblastosis induced by chronic hypoxia, which may lead to the suppression of platelet production in the bone marrow and result in the development of thrombocytopenia (Fandrey, 2004). Saxonhouse et al. (2006) have demonstrated that exposure to different levels of oxygen partial pressure (1, 5, or 20% oxygen for 10–12 days) decreases megakaryocyte colony formation. Bradford (2007) have proposed that decreased platelet production results from prolonged exposure to hypoxia, and short-term hypoxia increases platelet counts.

### Renal Angiogenesis

Angiogenesis is one of the most crucial processes involved in tissue repair and is considered an adaptive response to hypoxia. A study shows that in an *in vitro* model of hypoxia-injured kidneys, perfusable blood vessels within artificial tissue may be formed to provide oxygen and nutrients, and the vascular walls are subjected to a shear force, thus causing extension of the endothelial cells, which improves cell permeability (Kida et al., 2016). Based on the results of the present study, after 14 days of subjection under hypoxic conditions, all kidneys in the hypoxic groups presented with different degrees of angiogenesis, especially in group B. We believe that this effect is related to the expression level of VEGF because the expression level of VEGF was the highest in group B and corresponded to more active angiogenesis. Chade et al. (2018) have found that angiogenesis decreases when VEGF expression decreases. We believe that an increase in vascular density is associated with renal improvements. Logue et al. (2016) have reported that improved renal function is accompanied by increased microvascular density in vessels up to 80 μm in diameter, presumably via protection of the existing vasculature by VEGF. In contrast, VEGF is also a powerful proinflammatory mediator that can exacerbate the fibrotic response by promoting the extravasation of macrophages to the site of injury. Overexpression of VEGF contributes to capillary leakage and induces formation of non-functional blood vessels, thus aiding the creation of an anoxic microenvironment (Thurston et al., 1999; Thurston, 2002). Therefore, follow-up studies are warranted to explore the changes in the renal microenvironment.

### Inflammatory Reaction

There was a statistically significant difference between group B and groups C while inflammatory reaction was of no statistically significant difference between group A and group B. Previous studies have demonstrated that inflammatory factor can directly or indirectly promote angiogenesis (Ferrara et al., 2003; Li et al., 2008).

In theory, it is expected that greater active angiogenesis response in group C, however the findings in this study were contradictory. The most well-characterized molecular pathways that closely associate angiogenesis with hypoxia are the AKT/mTOR/HIF/VEGF and the TLR-4/NF-kB. The TLR-4/NF-kB signaling pathway is one of the most important signaling pathways in regulating various downstream inflammatory factors and participate in the regulation of inflammatory responses (Tian et al., 2019). Excessive secretion of pro-inflammatory cytokines is associated with exacerbated kidney injury (Jiang et al., 2020). It is clear that HIF-1α regulates VEGF protein synthesis through the PI3K pathway and the hypoxia-activated PI3K/Akt/mTOR pathway (Park et al., 2015). Moreover, the AKT/mTOR/HIF/VEGF is not only tightly controlled by positive and negative regulatory mechanisms but also closely coordinated with the TLR-4/NF-kB signaling pathway (Mongre et al., 2014). Many studies have shown that the suppression of NF-kB, the up-regulation of erythropoietin secretion (Nabavi et al., 2015). Unfortunately, we were not able to perform a higher molecular study in order to clarify the origin of this phenomenon.

In summary, we describe renal morphological alterations observed under different degrees of hypoxia and changes in the expression levels of HIF-1α, VEGF and inflammation factor and in the extent of angiogenesis that contribute to the kidney protection mechanism. Severe hypoxia may affect VEGF and expression and disrupt the balance of renal self-repair.

### Limitation

Rats may be exposed to normal air during feeding/cleaning (10 min every 48 h) and during the surgical operation (30 min maximum). This exposure cannot be ruled out and may have affected our results.

## Data Availability Statement

The original contributions presented in the study are included in the article/supplementary material, further inquiries can be directed to the corresponding author/s.

## Ethics Statement

This study was conducted in strict accordance with the recommendations of the guidelines of the Institutional Animal Care and Use Committee of Xinhua Hospital Affiliated with Shanghai Jiao Tong University School of Medicine (Shanghai, China), and the experimental protocol was approved by the Ethics Committee of the same institution (XHEC-F-2021-001).

## Author Contributions

YX and XK: study design. JL and TC: data collection. YW, JX, and YZ: statistical analysis. XZ: data interpretation. YX and XK: manuscript preparation. YZ: literature search. All authors contributed to the article and approved the submitted version.

## Conflict of Interest

The authors declare that the research was conducted in the absence of any commercial or financial relationships that could be construed as a potential conflict of interest.

## Abbreviations

ANOVA: analysis of variance
BE: buffer excess
CKD: chronic kidney disease
EPO: erythropoietin
HIF.: hypoxia-inducible factor
H&E: hematoxylin and eosin
Hb: hemoglobin
Hct: haematocrit
IOD: integrated optical density
PaO_2,_: arterial partial pressure of oxygen
PaCO_2,_: arterial partial pressure of carbon dioxide
PAS: Periodic acid-Schiff
PHD: prolyl hydroxylases
RBC: red blood cell
SCr: serum creatinine
SD: standard deviation
VEGF: vascular endothelial growth factor
VEGFR: vascular endothelial growth factor receptor.
